# Trends in Postacute Care Use and Outcomes After Hip and Knee Replacements in Dual-Eligible Medicare and Medicaid Beneficiaries, 2013-2016

**DOI:** 10.1001/jamanetworkopen.2020.0368

**Published:** 2020-03-04

**Authors:** Yue Li, Meiling Ying, Xueya Cai, Yeunkyung Kim, Caroline Pinto Thirukumaran

**Affiliations:** 1 Division of Health Policy and Outcomes Research, Department of Public Health Sciences, University of Rochester Medical Center, Rochester, New York; 2 Department of Biostatistics and Computational Biology, University of Rochester Medical Center, Rochester, New York; 3 Department of Orthopaedics, University of Rochester Medical Center, Rochester, New York

## Abstract

**Importance:**

Several Medicare alternative payment models were implemented in recent years, but their implications for socioeconomic gaps in postacute care (PAC) are unknown.

**Objectives:**

To determine the longitudinal trends in PAC use and outcomes after hip and knee replacements and in gaps among 3 groups: Medicare-only patients, dual-eligible patients with full Medicaid benefits, and dual-eligible patients with partial Medicaid benefits.

**Design, Setting, and Participants:**

A cohort study was conducted of PAC use and outcomes among Medicare fee-for-service patients undergoing hip or knee replacement surgery from January 1, 2013, to December 31, 2016, in approximately 3000 hospitals, using Medicare claims, assessment, hospital, and skilled nursing facility (SNF) files. Statistical analysis was performed from October 1, 2018, to December 17, 2019.

**Main Outcomes and Measures:**

Risk-adjusted differences among dual-eligible groups in institutional PAC use (SNF, inpatient rehabilitation, or long-term hospital care), readmission rate, and payment for readmissions; for patients discharged to a SNF, risk-adjusted differences in SNF quality measured by star ratings, proportion successfully discharged to the community, proportion transitioned to long-stay residence, and SNF length of stay and payments.

**Results:**

The sample included 1 302 256 patients (837 256 women [64.3%]; mean [SD] age, 75.4 [7.2] years) who underwent joint replacement. The proportion of patients discharged to institutional PAC and the 30-day and 90-day readmission rates decreased for all 3 groups during the period from 2013 to 2016. In 2013, institutional PAC use was 43.7% (95% CI, 43.5%-43.9%) for Medicare-only patients (n = 1 182 555), 70.1% (95% CI, 69.4%-70.8%; n = 60 461) for dual-eligible patients with full benefits, and 70.3% (95% CI, 69.6%-71.0%; n = 59 240) for dual-eligible patients with partial benefits; in 2016, the rates decreased to 32.5% (95% CI, 32.4%-32.7%) for Medicare-only patients, 62.3% (95% CI, 61.5%-63.0%) for dual-eligible patients with full benefits, and 61.5% (95% CI, 60.7%-62.3%) for dual-eligible patients with partial benefits. Among patients discharged to SNFs, outcomes remained flat over time. For example, the proportion of patients successfully discharged to the community remained at 80.5% (95% CI, 80.4%-80.7%) for Medicare-only patients, 59.8% (95% CI, 59.3%-60.3%) for dual-eligible patients with full benefits, and 50.0% (95% CI, 49.4%-50.5%) for dual-eligible patients with partial benefits. Multivariable analyses with adjustment for patient, hospital (or SNF), and geographical covariates suggested maintained or enlarged gaps in all outcomes.

**Conclusions and Relevance:**

This study suggests that, during the period from 2013 to 2016, Medicare patients undergoing hip or knee replacement showed reduced institutional PAC use, reduced readmissions, and, among those discharged to SNFs, roughly unchanged outcomes. However, dual-eligible patients, especially those with partial Medicaid benefits, had persistently worse outcomes than Medicare-only patients.

## Key Points

**Question:**

What are the longitudinal trends in the use of postacute care after hip and knee replacement surgery, and in gaps among Medicare patients of different socioeconomic status, under current Medicare payment reforms?

**Findings:**

This cohort study of 1.3 million Medicare patients who underwent joint replacement found reduced institutional postacute care use, reduced 30-day and 90-day readmission rates, and, among those discharged to a skilled nursing facility, roughly unchanged outcomes during the period from 2013 to 2016. However, Medicare and Medicaid dual-eligible patients had worse outcomes than Medicare-only patients over time.

**Meaning:**

Future Medicare reforms should be revised to align financial incentives with improved outcomes and equality of postacute and skilled nursing facility care.

## Teaser

This cohort study examines the longitudinal trends in postacute care use and outcomes after hip and knee replacements and in gaps among 3 groups: Medicare-only patients, dual-eligible patients with full Medicaid benefits, and dual-eligible patients with partial Medicaid benefits.

## Introduction

Hip and knee replacements are effective treatment options to improve physical function, pain relief, and quality of life for patients with advanced osteoarthritis. Hip and knee replacements are the most common inpatient procedures performed for older Medicare beneficiaries (eg, >400 000 procedures in 2014)^1^ and have been recently targeted by Medicare alternative payment models for improved quality and efficiency of care.^1,2,3^

Although most patients are discharged home after hip and knee replacements (possibly with some form of home health care), about 30% are discharged to postacute care (PAC) facilities, most commonly skilled nursing facilities (SNFs), to help optimize functional recovery.^4^ Postacute care practices after replacement procedures vary substantially^4,5,6,7^ owing to lack of consensus on the optimal setting and intensity of PAC, and owing to different Medicare reimbursement approaches for alternative PAC. Correspondingly, Medicare PAC spending and outcomes after joint replacements vary substantially.^7^ Studies on patients who have undergone hip or knee replacement who were discharged to SNFs further revealed that important outcomes, such as SNF length of stay (LOS), also vary considerably across SNFs with different levels of quality of care.^8^

Medicare beneficiaries who also receive Medicaid benefits (ie, dual-eligible beneficiaries) are characterized by low income, poor health, complex clinical and social needs, and high health care expenditures.^9^ They are thus highly vulnerable to issues such as uncoordinated care, unaffordable out-of-pocket expenditures, geographical distance from high-quality hospitals or PAC facilities, and less robust social support.^9,10,11^ Compared with non-Medicaid patients, Medicaid patients undergoing replacement procedures have higher inpatient resource use and worse outcomes, such as more postoperative complications.^12,13,14,15,16,17^ However, to our knowledge, research to date has not evaluated the possible disparities in PAC use and outcomes faced by Medicare-Medicaid dual-eligible patients after hip or knee replacements, particularly in the context of current Medicare payment reforms.

This study determined the national patterns of PAC use after hip and knee replacements from 2013 to 2016 and the variations among 3 groups of patients: Medicare-only patients, dual-eligible patients with full Medicaid benefits (ie, coverages for long-term care services and most Medicare cost-sharing and premium expenses), and dual-eligible patients with partial Medicaid benefits (ie, support for Medicare cost-sharing and/or premiums only).^11^ We further focused on patients discharged to SNFs and tracked gaps in several SNF-related outcomes.

## Methods

The research subjects review board at the University of Rochester Medical Center approved this study with a waiver of informed consent, because seeking informed consent from all patients included in the study was not feasible and the risk to study participants was minimal. This study followed the Strengthening the Reporting of Observational Studies in Epidemiology (STROBE) reporting guideline.

### Data and Samples

We analyzed the national Master Beneficiary Summary Files, which contain information on Medicare beneficiary enrollment status, demographic characteristics, and chronic conditions; Medicare Provider Analysis Review claims, which include records of care in acute-care hospitals, SNFs, and long-term care hospitals (LTCHs) for all fee-for-service Medicare beneficiaries; and several PAC assessment files, including the nursing home Minimum Data Sets, which contain detailed assessment and diagnosis data on patients in Medicare-certified SNFs, Inpatient Rehab Facility–Patient Assessment Instrument files, which contain detailed assessment and diagnosis data on patients in inpatient rehabilitation facilities (IRFs), and home health Outcome and Assessment Information Sets, which contain detailed assessment and diagnosis data on patients receiving Medicare-certified home health care. We also used the publicly available “Hospital Compare” files and hospital impact files to define hospital characteristics and “Nursing Home Compare” and LTCFocus files^18^ to define SNF characteristics. All data are for January 1, 2013, through December 31, 2016.

Our sample consisted of all discharges of Medicare fee-for-service beneficiaries who received total or partial hip or total knee replacement (Medicare severity–diagnosis related group 469 or 470, confirmed with *International Classification of Diseases* procedure codes), who were 65 years or older, who did not die or leave against medical advice at discharge, and whose initial admissions were between January 1 and October 1 of each study year (admissions after October 1 were excluded to ensure complete tracking of readmissions each year).

### Outcomes

The outcomes for all patients undergoing hip or knee replacement included whether the patient was discharged to institutional PAC (ie, SNF, IRF, or LTCH) and whether the patient was rehospitalized within 30 days of hospital discharge (30-day readmissions), total payments (ie, payments by Medicare, other third-party payers, and out of pocket) for all 30-day readmissions, and 90-day readmissions and corresponding total payments. Payments were inflation adjusted to 2016 dollars. Discharges to a specific PAC setting were identified as PAC uses with admission dates within 3 days of hospital discharge according to linked hospital claims and PAC assessment or claims files. Readmissions and payments for readmissions were defined using hospital claims.

In analyses of patients discharged to SNFs, we included several SNF-specific outcomes: whether the patient was discharged to a SNF with 4 or 5 stars, SNF LOS, total payments for the SNF stay (≤100 days), whether the patient was successfully discharged to the community after the SNF stay, and whether the patient became a long-stay resident. See the eAppendix in Supplement for more details.

### Independent Variables

Medicare and Medicaid dual-eligible status of beneficiaries was defined using the state-reported dual status indicator available in the Master Beneficiary Summary Files file.^19^ Two binary indicators were used to determine whether the beneficiary met the state income and resource criteria for full Medicaid benefits (ie, coverages of long-term care services and most Medicare cost-sharing and premium expenses) or only partial Medicaid benefits that provide support for prescription drug coverage only and/or premiums or copayments for Medicare services. The indicators were interacted with years (defined with a set of indicators) when estimating trends in differences.

### Covariates

Our analyses adjusted for patient, hospital, county, and state characteristics important to postdischarge outcomes.^4,5,6,7,12,13,14,15,16,20,21,22^ Patient covariates included demographic characteristics (age, sex, and race/ethnicity defined as non-Hispanic white, black, Hispanic, and other using 4 indicators according to information in the Master Beneficiary Summary Files) and indicator variables for the diagnosis of hip fracture and the presence of 31 comorbidities.^23^ Hospital covariates included indicators for profit status (for-profit, nonprofit, or government-owned), number of beds (3 indicators for small [<200 beds], medium [200-400 beds], and large [>400 beds]), medical school affiliation (yes or no), disproportionate patient percentage (a marker of caring for low-income patients),^24^ a case-mix index used by the Centers for Medicare & Medicaid Services for prospective payment, percentages of Medicaid patients and of black patients, and annual volume of hip and knee replacements. Additional covariates included rural or urban county of hospital location,^25^ county-level market competition measured by the Herfindahl-Hirschman index^26^ using number of hospital beds, and separate sets of indicators for states and years.

Analyses of patients who underwent hip or knee replacement and were discharged to SNFs were similarly adjusted for important patient, SNF, and geographic covariates.^3,5,8,27,28,29^ Patient covariates obtained from Minimum Data Sets admission assessments and Master Beneficiary Summary Files included demographic characteristics, marital status (married or not), whether the patient needed an interpreter for communication with health care professionals, difficulties in activities of daily living, cognitive function, depressive symptoms, and the presence of a set of chronic conditions. See the eAppendix in the Supplement for more details.

Skilled nursing facility covariates included number of beds; occupancy rate; chain affiliation (yes or no); profit status (for-profit, nonprofit, or government-owned); hospital affiliation (yes or no); percentages of Medicare residents and Medicaid residents among all current residents; nurse staffing levels for registered nurses, licensed practical nurses, and certified nursing assistants; and number of health care deficiency citations. Additional covariates included a county-level measure of market competition, urban or rural county of SNF location, and separate sets of indicators for states and years.

### Statistical Analysis

Statistical analysis was performed from October 1, 2018, to December 17, 2019. This study used separate patient-level, multivariable generalized linear models to determine the independent associations of dual-eligible status with outcomes of interest. A 2-sided *P* ≤ .05 was considered statistically significant. We first conducted bivariate analyses to examine differences in outcomes and in patient, hospital, and SNF characteristics in dual-eligible groups, using analyses of variance for continuous variables and χ^2^ tests for categorical variables. All regression models had the dual-eligible indicators as independent variables—each interacted with the 3-year indicators for 2014, 2015, and 2016—and adjusted for covariates. A joint *F* test on each set of 3 interactions was used to test for trends in disparities. See the eAppendix in the Supplement for more details on multivariable regression.

## Results

Our sample included 1 302 256 Medicare fee-for-service patients (837 256 women [64.3%]; mean [SD] age, 75.4 [7.2] years) undergoing hip or knee replacement during the period from 2013 to 2016, among whom 1 182 555 (90.8%) were Medicare-only beneficiaries, 60 461 (4.6%) were dual-eligible patients with full benefits, and 59 240 (4.5%) were dual-eligible patients with partial benefits. Compared with Medicare-only patients, dual-eligible patients were slightly older (mean [SD] age: Medicare-only patients, 75.1 [6.9] years; dual-eligible patients with full benefits, 75.8 [7.6] years; dual-eligible patients with partial benefits, 77.2 [8.4] years); less likely to be non-Hispanic white (Medicare-only patients, 92.3%; dual-eligible patients with full benefits, 64.7%; dual-eligible patients with partial benefits, 79.6%); more likely to have congestive heart failure (Medicare-only patients, 5.6%; dual-eligible patients with full benefits, 10.3%; dual-eligible patients with partial benefits, 12.5%), uncomplicated diabetes (Medicare-only patients, 15.9%; dual-eligible patients with full benefits, 24.9%; dual-eligible patients with partial benefits, 22.0%), complicated diabetes (Medicare-only patients, 4.1%; dual-eligible patients with full benefits, 8.0%; dual-eligible patients with partial benefits, 7.4%), and renal failure (Medicare-only patients, 9.3%; dual-eligible patients with full benefits, 12.5%; dual-eligible patients with partial benefits, 15.2%); and more likely to be discharged from hospitals with lower volumes for replacement surgery (mean [SD]: Medicare-only patients, 317.8 [334.0]; dual-eligible patients with full benefits, 223.1 [276.0]; dual-eligible patients with partial benefits, 217.8 [228.2]) (eTable 1 in the Supplement). Results in eTable 2 in the Supplement show similar differences by dual-eligible status among patients in SNFs.

**Figure 1.**
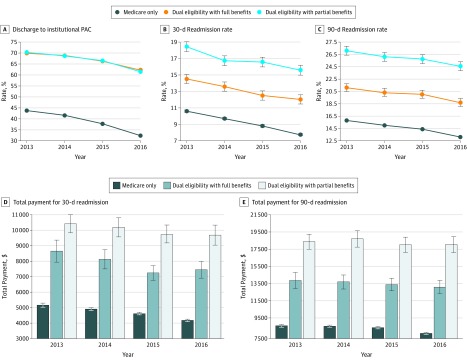
Discharge Destination, Readmission Rate, and Total Payment for Readmissions for Medicare Patients Undergoing Hip or Knee Replacement Surgery, by Dual-Eligible Status A, Discharge to institutional postacute care (PAC) vs discharge home with or without home health care. B, 30-Day readmission rate. C, 90-Day readmission rate. D, Total payments for 30-day readmission. E, Total payments for 90-day readmission. Error bars indicate 95% CIs.

During the period from 2013 to 2016, patients who underwent hip or knee replacement showed decreasing rates of institutional PAC discharge and readmissions (Figure 1). Among Medicare-only patients, 43.7% (95% CI, 43.5%-43.9%) were discharged to institutional PAC (35.3% to SNFs, 8.0% to IRFs, and 0.4% to LTCHs) in 2013, which decreased to 32.5% (95% CI, 32.4%-32.7%) in 2016 (27.4% to SNFs, 4.8% to IRFs, and 0.3% to LTCHs). Among dual-eligible patients with full or partial benefits, approximately 70% (full-benefit patients, 70.1%; 95% CI, 69.4%-70.8%; and partial-benefit patients, 70.3%; 95% CI, 69.6%-71.0%) were discharged to institutional PAC (58.8% to SNFs, 11.0% to IRFs, and 0.5% to LTCHs) in 2013, which decreased to 62.3% (95% CI, 61.5%-63.0%) for full-benefit patients) and 61.5% (95% CI, 60.7%-62.3%) for partial-benefit patients (54.0% to SNFs, 7.5% to IRFs, and 0.4% to LTCHs) in 2016. Correspondingly, the home-discharge rate (with or without home health care) increased for all groups during the period from 2013 to 2016 (from 56.4% to 67.5% for Medicare-only patients and from 29.8% to 38.1% for both dual-eligible groups). Unadjusted 30-day readmission rates decreased from 10.6% (95% CI, 10.5%-10.7%) in 2013 to 7.8% (95% CI, 7.7%-7.9%) in 2016 for Medicare-only patients, from 14.6% (95% CI, 14.0%-15.1%) to 12.0% (95% CI, 11.5%-12.6%) for dual-eligible patients with full benefits, and from 18.4% (95% CI, 17.8%-19.1%) to 15.6% (95% CI, 15.0%-16.2%) for dual-eligible patients with partial benefits.

These gaps by dual-eligible status either maintained or increased over time after adjusting for patient, hospital, and geographic factors (Table 1). For example, within 90 days of hospital discharge and compared with Medicare-only patients, dual-eligible patients with partial benefits were 24% (in 2013) to 37% (in 2016; *P* = .006 for trend in disparity) more likely to be readmitted (odds ratio, 1.24 [95% CI, 1.19-1.31] in 2013 and 1.37 [95% CI, 1.31-1.44] in 2016; *P* < .001 for both). Results in eTable 3 in the Supplement further suggested that a relatively large portion of these gaps were explained by chronic conditions (sequentially adjusted for in model 2) but not by demographic, hospital, or geographic factors.

**Table 1.** Adjusted Estimates of Postdischarge Outcomes for Dual-Eligible Medicare Patients Undergoing Hip or Knee Replacement^a^

Characteristic	Estimate (95% CI)			
	Dual-Eligible Patients With Full Benefits^b^	*P* Value for Trend Over Time	Dual-Eligible Patients With Partial Benefits^b^	*P* Value for Trend Over Time
Discharge to institutional PAC (vs discharge home with or without home health care)^c^				
2013	2.19 (2.07 to 2.32)	<.001	1.77 (1.68 to 1.87)	<.001
2014	2.28 (2.16 to 2.41)		1.83 (1.74 to 1.92)	
2015	2.40 (2.28 to 2.53)		1.99 (1.89 to 2.09)	
2016	2.59 (2.44 to 2.75)		2.06 (1.97 to 2.16)	
30-d Readmission^c^				
2013	0.91 (0.85 to 0.97)	.008	1.07 (1.01 to 1.14)	<.001
2014	0.93 (0.87 to 0.99)		1.05 (1.00 to 1.11)	
2015	0.93 (0.88 to 0.99)		1.16 (1.10 to 1.23)	
2016	1.04 (0.98 to 1.11)		1.25 (1.18 to 1.33)	
90-d Readmission^c^				
2013	1.01 (0.95 to 1.06)	.24	1.24 (1.19 to 1.31)	.006
2014	1.01 (0.96 to 1.07)		1.26 (1.20 to 1.31)	
2015	1.05 (0.99 to 1.10)		1.30 (1.25 to 1.37)	
2016	1.08 (1.02 to 1.14)		1.37 (1.31 to 1.44)	
Total payment for 30-d readmissions, $^d^				
2013	−205.64 (−627.84 to 216.56)	.01	830.53 (454.70 to 1206.36)	.32
2014	−280.12 (−661.54 to 101.29)		932.91 (547.24 to 1318.59)	
2015	−533.21 (−846.25 to −220.17)		903.77 (553.21 to 1254.34)	
2016	223.90 (−158.57 to 606.37)		1315.42 (905.73 to 1725.11)	
Total payment for 90-d readmissions, $^d^				
2013	24.18 (−547.60 to 622.96)	.43	3083.90 (2482.64 to 3685.17)	.55
2014	40.69 (−509.83 to 591.22)		3237.23 (2615.72 to 3858.73)	
2015	−102.97 (−602.22 to 396.28)		2923.87 (2343.40 to 3504.34)	
2016	479.53 (−85.09 to 1044.16)		3529.15 (2904.58 to 4153.72)	

Abbreviation: PAC, postacute care.

^a^ Multivariable logistic (for institutional PAC discharge and readmissions) and zero-inflated negative binomial (for readmission payments) regression models adjusted for patient, hospital, and geographical covariates listed in eTable 1 in the Supplement, as well as for time trends, state dummies, and clustering of patients in hospitals.

^b^ Reference group: Medicare-only patients.

^c^ Odds ratios are shown for these categories.

^d^ Estimated mean differences are shown for these categories.

**Figure 2.**
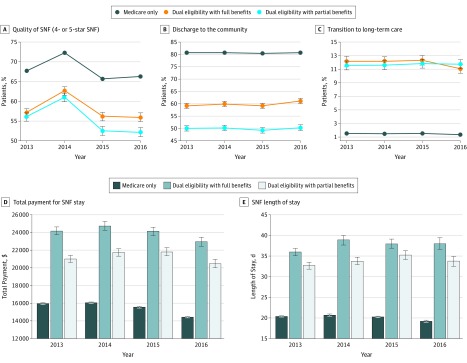
Quality of Admitting Skilled Nursing Facility (SNF), SNF Outcomes, and Total Payments for Patients Undergoing Hip or Knee Replacement Surgery Discharged to SNF, by Dual-Eligible Status A, Discharge to 4-star or 5-star SNF vs 1-star, 2-star, or 3-star SNF. B, Successful discharge to the community after SNF stay. C, Transition to long-term care after SNF stay. D, Total payment for SNF stay. E, Total SNF length of stay. Error bars indicate 95% CIs.

During the period from 2013 to 2016, among patients who underwent hip or knee replacement and were discharged to SNFs, 65.7% to 72.3% of Medicare-only patients were discharged to 4-star or 5-star SNFs, while 56.2% to 62.6% of dual-eligible patients with full benefits and 52.5% to 60.9% dual-eligible patients with partial benefits were discharged to 4-star or 5-star SNFs (Figure 2). During the period from 2013 to 2016, the unadjusted proportion of patients successfully discharged to community after a SNF stay was flat at 80.5% (95% CI, 80.4%-80.7%) for Medicare-only patients, 59.8% (95% CI, 59.3%-60.3%) for dual-eligible patients with full benefits, and 50.0% (95% CI, 49.4%-50.5%) dual-eligible patients with partial benefits. The unadjusted proportion of patients who transitioned to long-term residents after a SNF stay was 1.5% (95% CI, 1.4%-1.5%) for Medicare-only patients, 11.9% (95% CI, 11.6%-12.2%) for dual-eligible patients with full benefits, and 11.7% (95% CI, 11.3%-12.0%) for dual-eligible patients with partial benefits. The mean (SD) SNF LOS was 20.0 (21.1) days for Medicare-only patients, 38.0 (51.8) days for dual-eligible patients with full benefits, and 33.8 (44.4) days for dual-eligible patients with partial benefits.

Differences in SNF outcomes by dual-eligible status either maintained or increased over time after adjusting for patient, SNF, and geographical factors (Table 2). Compared with Medicare-only patients in a SNF, dual-eligible patients with full or partial benefits were half as likely to be successfully discharged to community (dual-eligible patients with full benefits, 2014: odds ratio, 0.51 [95% CI, 0.48-0.54]; dual-eligible patients with partial benefits, 2016: odds ratio, 0.44 [95% CI, 0.42-0.47]; *P* < .001), which persisted over time. Also, compared with Medicare-only patients, dual-eligible patients with full benefits had a longer SNF LOS by approximately 12 to 15 days (estimated mean difference, 11.87 days in 2013 and 15.80 days in 2016; *P* < .001 in both cases; *P* < .001 for trend), and dual-eligible patients with partial benefits had a longer SNF LOS by approximately 5 to 8 days (estimated mean difference, 5.02 days in 2013 and 7.93 days in 2016; *P* < .001 in both cases; *P* < .001 for trend). Results of eTable 4 in the Supplement further suggested that, with the exception of discharge to 4-star or 5-star SNFs, a relatively large portion of the differences in SNF outcomes by dual-eligible status were explained by physical function, cognition, and diagnoses (sequentially adjusted in model 2), but not by other covariates.

**Table 2.** Adjusted Estimates of SNF 5-Star Rating, SNF Outcomes, and Total Payments for SNF Stay for Dual-Eligible Medicare Patients Undergoing Hip or Knee Replacement and Discharged to SNF^a^

Characteristic	Estimate (95% CI)			
	Dual-Eligible Patients With Full Benefits^b^	*P* Value for Trend Over Time	Dual-Eligible Patients With Partial Benefits^b^	*P* Value for Trend Over Time
Discharge to 4-star or 5-star SNF (vs 1-star, 2-star, or 3-star SNF)^c^				
2013	0.89 (0.81-0.98)	.86	1.00 (0.92-1.09)	.01
2014	0.95 (0.85-1.06)		0.91 (0.83-0.98)	
2015	0.92 (0.83-1.03)		0.92 (0.84-1.00)	
2016	0.93 (0.85-1.03)		0.81 (0.75-0.89)	
Successful discharge to community^c^				
2013	0.49 (0.46-0.52)	.64	0.48 (0.46-0.51)	.21
2014	0.51 (0.48-0.54)		0.47 (0.45-0.50)	
2015	0.48 (0.45-0.52)		0.46 (0.43-0.49)	
2016	0.50 (0.47-0.53)		0.44 (0.42-0.47)	
Transition to long-term care after SNF stay^c^				
2013	5.11 (4.64-5.64)	.003	3.61 (3.26-4.00)	<.001
2014	5.70 (5.16-6.29)		3.91 (3.53-4.33)	
2015	5.92 (5.34-6.56)		4.07 (3.67-4.52)	
2016	6.69 (5.99-7.46)		4.99 (4.47-5.58)	
Total payment for SNF stay, $^d^				
2013	5801.69 (4440.81-5722.576)	.23	2149.48 (1799.84-2499.11)	<.001
2014	5621.85 (4993.73-6249.96)		2503.05 (2131.73-2874.36)	
2015	5441.81 (4971.11-5912.50)		3115.01 (2716.72-3513.30)	
2016	5555.35 (4997.83-6112.87)		3163.36 (2773.20-3553.51)	
SNF length of stay, d^d^				
2013	11.87 (10.96-12.78)	<.001	5.02 (4.25-5.79)	<.001
2014	14.61 (13.48-15.74)		6.17 (5.27-7.07)	
2015	14.23 (13.08-15.39)		7.99 (6.95-9.04)	
2016	15.80 (14.36-17.25)		7.93 (6.81-9.06)	

Abbreviation: SNF, skilled nursing facility.

^a^ Multivariable logistic (for discharge to 4-star or 5-star SNF, community discharge, and transition to long-term care) and negative binomial (for SNF payments and length of stay) regression models adjusted for patient, SNF, and geographical covariates listed in eTable 2 in the Supplement, as well as for time trends, state dummies, and clustering of patients in SNFs.

^b^ Reference group: Medicare-only patients discharged to SNF.

^c^ Odds ratios are shown for these categories.

^d^ Estimated mean differences are shown for these categories.

## Discussion

This national study of Medicare fee-for-service patients undergoing hip or knee replacement found decreasing trends in hospital discharge to institutional PAC settings and in 30-day and 90-day readmissions during the period from 2013 to 2016. Meanwhile, patients with dual eligibility for Medicare and Medicaid showed persistently higher rates of institutional PAC use and of readmission and higher total payments for readmissions. Among patients discharged to SNFs, SNF resource use and outcomes did not improve over time and were worse among dual-eligible patients. Multivariable analyses further suggested that differences in these outcomes by dual-eligible status either persisted or increased over time.

Previous studies showed that Medicaid patients had higher rates of inpatient resource use, higher costs, and higher rates of readmission after hip or knee replacement than non-Medicaid patients.^12,13,14,15,16,17^ However, research is lacking on PAC disparities after joint replacement surgery. This study contributes to the literature by demonstrating more intensive institutional PAC use but lower PAC quality and outcomes for dual-eligible patients relative to Medicare-only patients.

Recent Medicare payment reforms that align financial incentives with quality and efficiency of care may be associated with the reduced rates of institutional PAC use and readmissions in our study. For example, Medicare’s Hospital Readmissions Reduction Program started penalizing hospitals with excessively high 30-day readmission rates in October 2012. Evidence suggests that, although the Hospital Readmissions Reduction Program initially targeted only several medical conditions, it may have a beneficial spillover effect that helped reduce readmission rates for surgical patients, including those undergoing hip or knee replacement.^30,31,32^ Model 2 of the Medicare Bundled Payments for Care Improvement initiative, which started in 2013 and encouraged hospitals to voluntarily participate in bundled payments for 1 or more of 48 episodes of care, was also shown to be associated with reduced rates of institutional PAC use and reduced duration of PAC after hip or knee replacement.^2^ Moreover, the Medicare Shared Savings Program, the most common type of Medicare Accountable Care Organization started in 2012, was designed to have different health care professionals deliver the full continuum of care to Medicare beneficiaries and be responsible for the quality and total costs of care under the Accountable Care Organization. Emerging evidence shows that hospitals and SNFs participating in the Medicare Shared Savings Program are successful in reducing PAC expenditures, institutional PAC use, and readmissions.^33,34,35^

Our results found similarly reduced rates of institutional PAC use and of readmission for the 3 patient groups by Medicaid eligibility status. Although reducing institutional PAC use may help reduce wasteful Medicare spending under current Medicare reforms, medically and socially vulnerable patients, including Medicaid-eligible patients, have been shown to have a higher demand for inpatient rehabilitation and other institutional PAC after lower extremity joint replacement^36^ because these patients are usually more functionally impaired, have more medical conditions, lack adequate access to outpatient rehabilitation services, and have less appropriate social and community support.^9,10^ Thus, it is conceivable that dual-eligible patients undergoing hip or knee replacement are more likely to use institutional PAC as an important safety net for their postdischarge care, and current efforts to divert patients who need PAC from inpatient settings to home health care may inadvertently worsen disparities in postdischarge outcomes, such as risk of readmissions. This concern is somewhat supported by the adjusted results showing a persistent or increased gap over time in the 30-day and 90-day readmission rates (Table 1).

Dual-eligible patients are heterogeneous, and those who qualified partially for Medicaid benefits in this study seemed to be the most vulnerable, showing the highest readmission rate and lowest chance of successful community discharge after SNF stay. Although dual-eligible patients seldom leave the Medicare program, their Medicaid benefits are more volatile owing to reasons such as changes in income or assets and failure to apply for renewal of Medicaid eligibility.^9^ Medicaid patients with partial benefits tend to have slightly higher incomes than those with full benefits (because of Medicaid’s stricter income eligibility criteria for the latter); however, it has been shown that loss of Medicaid coverage (typically for several months in a year) is more common among those with partial benefits than among those with full benefits and that those who lose Medicaid coverage typically have no other supplemental insurance.^9,11^ Loss of Medicaid coverage thus tends to be associated with increased liability for Medicare cost-sharing and premiums, compromised access to care, and disrupted continuity of care. Our results of persistently higher readmission rates and worse SNF outcomes for dual-eligible patients with partial benefits highlight these issues for them relative to other dual-eligible and Medicare-only patients.

Skilled nursing facilities are high-cost, high-volume PAC facilities, accounting for the greatest part of total PAC spending. The quality and outcomes of SNF care, however, are often inadequate and vary substantially across facilities and patient groups.^8,27,28,29^ Nevertheless, with the exception of readmission rate, the aforementioned Medicare alternative payment models do not directly incentivize SNFs to improve their quality and outcomes. For example, under the Medicare Shared Savings Program, health care professionals in Medicare Accountable Care Organizations have to meet 31 quality benchmarks to receive rewards and avoid financial penalties; these quality metrics cover experiences with ambulatory care according to a standard survey of patients, care coordination and patient safety measured by readmissions (relevant to SNF and other patients with recent hospitalization) and other indicators, preventive care (eg, colorectal cancer screening), and care for at-risk populations (eg, patients with diabetes).^37^ No quality metrics under the Medicare Shared Savings Program focus on SNF outcomes. Moreover, a recent Medicare bundled payments program targeting hip and knee replacement, the Comprehensive Care for Joint Replacement model that began April 2016, focuses on 2 sets of measures (postoperative complications and experience with hospital care) that largely ignore PAC and SNF outcomes.^1^

The lack of attention by recent Medicare reforms to SNF performance may underlie the flat trends in SNF outcomes in our study (Figure 2). However, outcomes such as successful discharge to the community are patient-centered, and for patients with joint replacement, being able to function independently in the community is the fundamental goal of the procedure. Perhaps even more concerning are the parallel findings of gaps among patient groups; although 80.5% of Medicare-only patients who underwent hip or knee replacement had a successful community discharge after SNF stay, the corresponding proportions were between 50.0% and 59.8% for dual-eligible patients; and although 1.5% of Medicare-only patients became long-term nursing home residents after staying at SNFs, the proportions were between 11.7% and 11.9% for dual-eligible patients. Together, these findings suggest an urgent need for future Medicare reforms to incorporate incentives for improved outcomes of SNF care, especially for vulnerable patients such as dual-eligible patients.

### Limitations

This study had several limitations. First, because this study was limited to Medicare fee-for-service patients, our conclusions may or may not be generalized to Medicare Advantage patients. Second, our study relied on administrative data and was unable to evaluate other outcomes, such as functional status and quality of life after joint replacement. Third, although our analyses adjusted for a set of patient, institution, and geographic covariates, it is possible that differences by dual-eligible status were partially mediated by unmeasured factors such as availability of community-based support.

## Conclusions

This study found that during the period from 2013 to 2016, Medicare patients undergoing hip or knee replacement showed reduced rates of institutional PAC use and reduced rates of 30-day and 90-day readmission. Among patients discharged to SNFs, important SNF outcomes did not improve over time. Medicare and Medicaid dual-eligible patients had persistently higher rates of institutional PAC use, higher readmission rates, and worse SNF outcomes than Medicare-only patients, with gaps either being maintained or increasing over time. Future Medicare reforms should align financial incentives with improved outcomes and equality of SNF care.

## Notes

Supplement. **eAppendix.** SNF-Specific Outcome Measures **eTable 1.** Characteristics of Medicare Patients Undergoing Hip/Knee Replacement Surgery in 2016, by Dual Eligibility Status **eTable 2.** Characteristics of Medicare Patients Undergoing Hip/Knee Replacement Surgery and Discharged to Skilled Nursing Facility in 2016, by Dual Eligibility Status **eTable 3.** Postdischarge Outcomes for Dually Eligible Medicare Patients Undergoing Hip/Knee Replacement in 2016, With Sequential Adjustment **eTable 4.** Sequentially Adjusted Estimates of Skilled Nursing Facility (SNF) 5-Star Rating, Payment for SNF Stay, SNF Outcomes and Payments for Dually Eligible Medicare Patients Undergoing Hip/Knee Replacement and Discharged to SNF in 2016 **eReferences.**
