# Supplementary material for: Trends in Postacute Care Use and Outcomes After Hip and Knee Replacements in Dual-Eligible Medicare and Medicaid Beneficiaries, 2013-2016
Source: JAMA Netw Open. 2020 Mar 4;3(3):e200368. doi: 10.1001/jamanetworkopen.2020.0368 (PMC7057132; doi:10.1001/jamanetworkopen.2020.0368)
Supplement: Supplement. — eAppendix. SNF-Specific Outcome Measures eTable 1. Characteristics of Medicare Patients Undergoing Hip/Knee Replacement Surgery in 2016, by Dual Eligibility Status eTable 2. Characteristics of Medicare Patients Undergoing Hip/Knee Replacement Surgery and Discharged to Skilled Nursing Facility in 2016, by Dual Eligibility Status eTable 3. Postdischarge Outcomes for Dually Eligible Medicare Patients Undergoing Hip/Knee Replacement in 2016, With Sequential Adjustment eTable 4. Sequentially Adjusted Estimates of Skilled Nursing Facility (SNF) 5-Star Rating, Payment for SNF Stay, SNF Outcomes and Payments for Dually Eligible Medicare Patients Undergoing Hip/Knee Replacement and Discharged to SNF in 2016 eReferences. [file jamanetwopen-3-e200368-s001.pdf]

Supplementary Online Content

Li Y, Ying M, Cai X, Kim Y, Thirukumaran CP. Trends in postacute care use and outcomes after hip and knee replacements in dual-eligible Medicare and Medicaid beneficiaries, 2013-2016. *JAMA Netw Open.* 2020;3(3):e200368. doi:10.1001/jamanetworkopen.2020.0368

eAppendix. SNF-Specific Outcome Measures
eTable 1. Characteristics of Medicare Patients Undergoing Hip/Knee Replacement Surgery in 2016, by Dual Eligibility Status
eTable 2. Characteristics of Medicare Patients Undergoing Hip/Knee Replacement Surgery and Discharged to Skilled Nursing Facility in 2016, by Dual Eligibility Status
eTable 3. Postdischarge Outcomes for Dually Eligible Medicare Patients Undergoing Hip/Knee Replacement in 2016, With Sequential Adjustment
eTable 4. Sequentially Adjusted Estimates of Skilled Nursing Facility (SNF) 5-Star Rating, Payment for SNF Stay, SNF Outcomes and Payments for Dually Eligible Medicare Patients Undergoing Hip/Knee Replacement and Discharged to SNF in 2016
eReferences.

This supplementary material has been provided by the authors to give readers additional information about their work.

© 2020 Li Y et al. *JAMA Network Open.*

eAppendix. SNF-Specific Outcome Measures

The 5-star ratings of nursing homes were developed by the CMS and published on the "Nursing Home Compare" website to inform prospective patients of quality of care, and to foster facility quality improvement efforts.[1] The ratings were developed to summarize 3 domains of "quality": deficiency citations assigned to the facility during annual and complaint inspections; clinical outcomes of residents based on MDS assessments (e.g. prevalence of falls with major injury); and nurse staffing to resident ratios. The CMS aggregated measures of these 3 domains into a rating system from one to five stars, with more stars indicating better quality.[2] According to CMS definitions of published quality measures, SNF patients are considered to have a successful discharge to the community if they are discharge to the community within 100 days of entry, and do not die, have an unplanned inpatient admission, or enter/reenter a nursing home within 30 days of discharge to the community.[3] Finally, we defined SNF patients as being transitioned to long-term residents if they stayed in the nursing home longer than 100 days.

Patient covariates obtained from MDS admission assessment records

Analyses focusing on hip/knee replacement patients discharged to SNFs adjusted for important patient, SNF and other covariates.[4-9] Patient covariates obtained from MDS admission assessment records and MBSF files included demographics, whether the resident was currently married, whether the resident needed an interpreter for communication with healthcare providers, difficulties in activities of daily living (ADLs), cognitive function scale of the resident, the 9-item Patient Health Questionnaire (PHQ-9) score for depressive symptom, and the presence of a set of chronic conditions.

© 2020 Li Y et al. *JAMA Network Open.*

The 7-component ADL measure included bed mobility, transfer, dressing, eating, toilet use, personal hygiene, and bathing. We coded each component as 5 categories from 0 (independence) to 4 (total dependence), with the range of the aggregate ADL score being 0 to 28 (thus, higher score indicating more dependence). Resident cognitive function scale was defined based on the Brief Interview for Mental Status (BIMS)[10] score and, for residents not able to complete the BIMS assessments, the cognitive performance scale (CPS) according to staff assessments.[11] The cognitive function scale was coded on a scale of 0 to 3 (0 – being cognitively intact; 1 – mild impairment in cognition; 2 – moderate impairment; and 3 – severe impairment). The PHQ-9 (or a staff-observation version of it for residents who could not make themselves understood or could not complete the interview due to cognitive impairment) is currently used by the CMS to assess the depressive symptoms of all nursing home residents.[12] The validity and reliability of the PHQ-9 for use among nursing home residents have been established.[13,14] The PHQ-9 scale ranges from 0 to 27 with higher score indicating more severe depressive symptoms; residents with a score of 10 or higher are usually considered as at risk for clinically significant depression and will trigger an MDS-related care area assessment and the development of a targeted care plan for the resident.[12]

Details of statistical analysis (multivariable regression)

GLM assuming binomial distribution and with a logit link function were fit for binary outcomes (e.g., if the patient had any readmission within 30 days after hospital discharge), in which robust variance estimators accounted for the clustering of patients in hospital (or SNF).[15]

Total payments for 30-day and for 90-day readmissions among all hip/knee replacement patients were highly skewed with a large number of zeros (for patients not readmitted). Thus, we modeled each payment using zero-inflated negative binomial (ZINB) regression to account for the excessive number of zeros and overdispersion in payment.[16] The ZINB regression had two parts; the first part was a robust GLM with a logit link function and assuming binomial

© 2020 Li Y et al. *JAMA Network Open.*

distribution, which estimated the likelihood of any readmission, and the second part was a robust GLM assuming negative binomial distribution, which modeled the amount of total payment conditional on readmission. Models in both parts had dual eligible status as key independent variables (interacted with year indicators) and adjusted for all covariates. After model estimation we calculated the average effects of dual eligible status on total payment for readmissions. We further fit separate negative binomial models on total payment for SNF stay and SNF LOS (among patients discharged to SNF), and reported estimated mean differences of dual eligible status.

Finally, we fit the above models with the 2016 data that had dual eligible status as key independent variables, and that sequentially adjusted for no covariate (model 0); patient age, gender, and race/ethnicity (model 1); other patient covariates (model 2); hospital (or SNF) characteristics (model 3); and geographic covariates (model 4). The differential estimates in these models with sequential adjustments helped elucidate the degree to which alternative covariates underlay gaps in outcomes by dual eligible status; given this purpose, we limited the sequential analyses to the 2016 data to obviate possible confounding due to longitudinal changes in disparities (tested in previous analyses).

© 2020 Li Y et al. *JAMA Network Open.*

eTable 1. Characteristics of Medicare Patients Undergoing Hip/Knee Replacement Surgery in 2016, by Dual Eligibility Status

	Medicare only (n=320,801)*	Dually eligible – full (n=14,915)*	Dually eligible – partial (n=14,653)*
	Mean±SD or Prevalence (%)		
Age in years	75.1 (6.9)	75.8 (7.6)	77.2 (8.4)
Race and ethnicity, %			
White	92.3	64.7	79.6
Black	4.2	12.7	12.1
Hispanic	0.3	9.2	4.0
Other	3.2	13.4	4.3
Male gender, %	37.5	21.6	24.6
Admission type, %			
Elective	85.8	70.8	61.6
Emergent	9.2	21.1	29.0
Urgent	4.4	6.9	7.6
Other	0.6	1.2	1.8
Hip fracture	11.3	26.5	36.7
Elixhauser comorbidities, %			
Congestive heart failure	5.6	10.3	12.5
Cardiac arrhythmias	17.1	17.0	21.2
Valvular disease	5.8	5.1	7.2
Pulmonary circulation disorders	1.7	2.3	3.4
Peripheral vascular disorders	4.1	5.1	6.2
Hypertension, uncomplicated	64.0	67.3	64.0
Paralysis	0.1	0.3	0.3
Other neurological disorders	3.4	7.4	7.9
Chronic pulmonary disease	15.0	24.2	24.0
Diabetes, uncomplicated	15.9	24.9	22.0
Diabetes, complicated	4.1	8.0	7.4
Hypothyroidism	20.3	19.5	20.9
Renal failure	9.3	12.5	15.2
Liver disease	0.9	1.6	1.6
Peptic ulcer disease excluding bleeding	0.4	0.5	0.6
AIDS/HIV	0.0	0.1	0.1
Lymphoma	0.3	0.3	0.3
Metastatic cancer	0.4	0.2	1.0
Solid tumor without metastasis	1.0	0.9	1.8
Rheumatoid arthritis/Collagen vascular	4.5	5.4	5.2
Coagulopathy	3.2	4.0	4.8
Obesity	19.4	19.8	17.2
Weight loss	1.0	2.3	4.4
Fluid and electrolyte disorders	11.1	16.7	19.9
Blood loss anemia	0.9	1.5	1.5
Deficiency anemia	1.7	2.7	3.3
Alcohol abuse	1.2	1.4	1.8
Drug use	0.5	1.4	1.0

© 2020 Li Y et al. *JAMA Network Open.*

Psychoses	0.1	2.0	1.1
Depression	12.0	17.9	17.4
Hypertension, complicated	8.9	12.7	15.0
Ownership status of the hospital, %			
Government	10.4	11.2	14.0
Private for-profit	17.1	16.3	16.9
Private not-for-profit	72.5	72.5	69.1
Number of beds, %			
Small (<200beds)	38.9	36.9	41.0
Medium(>=200 & <400)	33.3	35.7	34.0
Large(>=400)	27.8	27.4	25.0
Medical school affiliation, %	47.6	51.2	46.5
Disproportionate patient percent	0.2 (0.1)	0.3(0.2)	0.3(0.1)
Transfer-adjusted casemix index	1.7(0.3)	1.7(0.3)	1.7(0.3)
Volume of hip/knee replacements	317.8(334.0)	223.1(276.0)	217.8(228.2)
Urban, %	90.9	88.2	83.5
Percent of Medicaid patients in hospital	0.2 (0.1)	0.4 (0.2)	0.3(0.1)
Percent of non-Hispanic Black patients in hospital	0.1(0.1)	0.1(0.1)	0.1(0.1)
Market competition[a]	0.6(0.3)	0.6(0.3)	0.5(0.3)

*P<0.01 for comparisons of all characteristics across patient groups, based on analyses of variance of variance for continuous variables and chi-square tests for categorical variables.

[a]According to Herfindahl-Hirschman Index, calculated as 1- the sum of squared shares of hospital beds for all hospitals in the county. Market competitiveness scores ranged from 0 (least competition) to 1(highest competition).

SD=standard deviation.

© 2020 Li Y et al. *JAMA Network Open.*

eTable 2. Characteristics of Medicare Patients Undergoing Hip/Knee Replacement Surgery and Discharged to Skilled Nursing Facility in 2016, by Dual Eligibility Status

	Medicare only (n=80,963)*	Dually eligible – full (n=7,842)*	Dually eligible – partial (n=7,465)*
	Mean±SD or Prevalence (%)		
Age in years	78.2(7.7)	77.1(8.2)	79.2(8.6)
Race and ethnicity, %			
White	92.1	68.4	83.8
Black	5.2	11.8	10.5
Hispanic	0.3	6.5	2.6
Other	2.4	12.3	3.1
Male gender, %	28.5	20.3	23.3
Marital status , %			
Not married	53.0	76.7	76.0
Married	43.7	19.8	20.7
Missing	3.3	3.5	3.4
Interpreter needed, %			
No	97.7	81.9	88.1
Yes	0.5	14.2	3.1
Missing	1.8	3.9	8.8
Activities of daily living at SNF admission(0-28)[a]	13.2(5.3)	14.8(5.4)	14.9(5.5)
Cognitive function scale[b], %			
0	71.9	54.9	47.0
1	16.0	21.4	21.6
2	4.8	12.1	12.5
3	0.3	0.8	0.5
Missing	7.0	10.8	28.4
PHQ-9 [c], %			
<10	85.8	79.4	68.5
>=10	1.2	1.5	1.8
Missing	13.0	19.1	29.7
Chronic diseases, %			
Acute myocardial infraction	5.5	6.2	9.0
Alzheimer's disease	25.3	46.8	51.3
Atrial Fibrillation	23.4	21.0	25.5
Cataract	76.5	76.1	74.0
Chronic kidney disease	42.4	52.1	56.1
Chronic obstructive pulmonary disease	31.8	46.4	46.8
Congestive heart failure	33.3	48.6	48.6
Diabetes	40.0	57.9	49.3
Glaucoma	26.7	30.3	25.7
Hip/pelvic fracture	30.3	42.7	56.7
Ischemic heart disease	56.4	68.1	65.7
Depression	47.9	68.0	65.5
Osteoporosis	35.7	45.7	41.5
Rheumatoid arthritis/osteoarthritis	93.3	93.2	87.9

© 2020 Li Y et al. *JAMA Network Open.*

Stroke/transient ischemic attack	18.6	25.8	26.1
Breast cancer	9.3	6.8	8.4
Colorectal cancer	3.4	3.5	4.2
Prostate cancer	5.4	2.8	3.8
Lung cancer	1.9	1.9	2.6
Endometrial cancer	1.7	1.9	2.3
Anemia	84.2	90.9	89.6
Asthma	21.6	31.4	27.0
Hyperlipidemia	86.8	87.3	85.6
Benign prostatic hyperplasia	19.0	13.2	14.9
Hypertension	92.1	95.8	95.1
Acquired hypothyroidism	37.3	40.2	38.6
Number of beds	118.7(71.0)	135.1(88.4)	126.3(70.8)
Nursing hours per resident day for			
Registered nurse	0.8(0.9)	0.7(0.8)	0.6(0.7)
Licensed practical nurse	0.9(0.6)	0.9(0.6)	0.9(0.5)
Certified nursing assistance	2.6(0.8)	2.5(0.7)	2.4(0.7)
Number of deficiency citations	5.6(5.2)	7.2(6.5)	6.5(5.8)
Percent of Medicaid patients in nursing home	37.5(25.7)	51.4(25.0)	52.5(23.3)
Percent of Medicare patients in nursing home	31.0(22.6)	23.9(20.2)	22.6(18.6)
Hospital-based, %	9.2	6.5	6.3
Profit status, %			
For-profit	59.3	71.4	68.3
Nonprofit	36.3	24.9	16.4
Government	4.4	3.7	5.3
Chain affiliation, %	56.2	54.3	59.8
Urban,%	86.2	82.3	75.7
Market competition[c]	0.86(0.17)	0.86(0.19)	0.81(0.22)

*P<0.01 for comparisons of all characteristics across patient groups, based on analyses of variance of variance for continuous variables and chi-square tests for categorical variables.

[a]Seven types of activities of daily living were coded 0 (independence) to 4 (total dependence) for a total score of 0-28.

[b]Coded 0 for being cognitively intact to 3 for severely impaired cognition.

[c]According to Herfindahl-Hirschman Index, calculated as 1- the sum of squared shares of nursing home beds for all nursing homes in the county. Market competitiveness scores ranged from 0 (least competition) to 1(highest competition).

SD=Standard Deviation; PHQ-9=9 item Patient Health Questionnarie.

© 2020 Li Y et al. *JAMA Network Open.*

eTable 3. Postdischarge Outcomes for Dually Eligible Medicare Patients Undergoing Hip/Knee Replacement in 2016, With Sequential Adjustment[a]

	Unadjusted rate or dollar amount	Model 0	Model 1	Model 2	Model 3	Model 4
		Adjusted Odds Ratio (95% Confidence Interval)				
**Discharge to institutional PAC (vs home discharge with or w/o home health), %**						
Medicare only	32.52%	Ref.	Ref.	Ref.	Ref.	Ref.
Dually eligible – full	62.27%	3.42 (3.23-3.62)	3.60 (3.39-3.83)	2.64 (2.48-2.81)	2.54 (2.40-2.69)	2.57 (2.43-2.73)
Dually eligible – partial	61.53%	3.32 (3.17-3.47)	2.98 (2.83-3.13)	1.98 (1.88-2.09)	1.94 (1.84-2.04)	1.97 (1.87-2.07)
**30-day readmission, %**						
Medicare only	7.77%	Ref.	Ref.	Ref.	Ref.	Ref.
Dually eligible – full	12.04%	1.62 (1.53-1.73)	1.58 (1.48-1.68)	1.16 (1.09-1.24)	1.06 (0.99-1.13)	1.06 (0.99-1.2)
Dually eligible – partial	15.63%	2.20 (2.08-2.32)	1.90 (1.80-2.01)	1.31 (1.23-1.38)	1.23 (1.16-1.30)	1.21 (1.14-1.28)
**90-day readmission, %**						
Medicare only	13.14%	Ref.	Ref.	Ref.	Ref.	Ref.
Dually eligible – full	18.75%	1.53 (1.45-1.61)	1.53 (1.46-1.62)	1.17 (1.10-1.23)	1.10 (1.04-1.16)	1.10 (1.04-1.16)
Dually eligible – partial	24.61%	2.16 (2.07-2.26)	1.99 (1.90-2.08)	1.43 (1.36-1.50)	1.38 (1.31-1.44)	1.36 (1.30-1.43)
**Total payment for 30-day readmissions, $**		Estimated mean difference (95% Confidence Interval)				
Medicare only	4201.45	Ref.	Ref.	Ref.	Ref.	Ref.
Dually eligible – full	7464.86	3263.41 (2667.09-3859.73)	2966.13 (2398.58-3533.39)	1188.17 (735.62-1640.72)	482.91 (93.65-872.17)	327.12(-41.18-695.41)
Dually eligible – partial	9713.73	5512.28 (4810.00-6214.56)	4249.25 (3647,60-4850.90)	1558.09 (1125.38-1990.79)	1209.34(809.64-1609.04)	1198.24(814.91-1581.57)
**Total payment for 90-day readmissions, $**						
Medicare only	8100.62	Ref.	Ref.	Ref.	Ref.	Ref.
Dually eligible – full	13321.14	5220.52 (4336.94-6104.10)	5137.59 (4260.52-6014.66)	2097.76 (1386.53-2808.99)	1030.86 (422.77-1638.95)	643.06(83.90-1202.23)
Dually eligible – partial	18077.84	9977.22 (8986.47-10967.96)	8697.40 (7784.61-9610.18)	3937.96 (3253.67-4622.26)	3436.22(2795.55-4076.89)	3484.09(2863.39-41-4.79)

Model 0: generalized linear model, zero-inflated negative binomial, or negative binomial model with no covariate adjustment;

Model 1: model 0, with further adjustment for age, gender, and race/ethnicity;

Model 2: model 1 with further adjustment for other patient characteristics;

Model 3: model 2, with further adjustment for hospital characteristics;

Model 4: model 3, with further adjustment for market competition, rural/urban location, and state dummies.

© 2020 Li Y et al. *JAMA Network Open.*

eTable 4. Sequentially Adjusted Estimates of Skilled Nursing Facility (SNF) 5-Star Rating, Payment for SNF Stay, SNF Outcomes and Payments for Dually Eligible Medicare Patients Undergoing Hip/Knee Replacement and Discharged to SNF in 2016[a]

	Unadjusted rate, dollar amount, or days	Model 0	Model 1	Model 2	Model 3	Model 4
		Adjusted Odds Ratio (95% Confidence Interval)				
**Discharge to 4- or 5-star SNF (vs 1-3 star SNF), %**						
Medicare only	66.27%	Ref.	Ref.	Ref.	Ref.	Ref.
Dually eligible – full	55.98%	0.65 (0.59-0.71)	0.64 (0.58-0.70)	0.72 (0.67-0.78)	1.01 (0.92-1.11)	0.93 (0.85-1.10)
Dually eligible – partial	52.23%	0.56 (0.52-0.60)	0.56 (0.52-0.61)	0.69 (0.65-0.74)	0.80 (0.74-0.86)	0.86 (0.80-0.93)
**Successful discharge to the community, %**						
Medicare only	80.57%	Ref.	Ref.	Ref.	Ref.	Ref.
Dually eligible – full	61.10%	0.38 (0.36-9.40)	0.28 (0.26-0.30)	0.49 (0.45-0.52)	0.49 (0.46-0.53)	0.49 (0.46-0.53)
Dually eligible – partial	50.29%	0.24 (0.23-0.26)	0.22 (0.21-0.24)	0.43 (0.41-0.46)	0.44(0.41-0.47)	0.44 (0.41-0.47)
**Transition to long-term residents after SNF stay, %**						
Medicare only	1.29%	Ref.	Ref.	Ref.	Ref.	Ref.
Dually eligible – full	11.03%	9.49 (8.56-10.52)	12.80 (11.51-14.23)	7.35 (6.54-8.27)	6.94 (6.16-7.83)	7.04 (6.23-7.95)
Dually eligible – partial	11.71%	10.15 (9.20-11.20)	10.21 (9.22-11.29)	5.40 (4.82-6.06)	5.19 (4.62-5.84)	5.21 (4.63-5.86)
**Total payment for SNF stay, $**		Estimated mean difference (95% Confidence Interval)				
Medicare only	14482.37	Ref.	Ref.	Ref.	Ref.	Ref.
Dually eligible – full	22989.37	8507.25 (7572.48-9442.02)	8970.22 (8102.16-9838.29)	5505.40 (4841.53-6169.27)	5851.60 (5189.75-6513.44)	5589.06 (4989.63-6188.48)
Dually eligible – partial	20527.29	6045.17 (5517.97-6572.36)	5502.74 (5009.06-5996.42)	2588.43 (2182.43-2994.43)	2829.35 (2436.00-3222.71)	2837.06 (2444.98-3229.15)
**SNF length of stay, days**						
Medicare only	19.22	Ref.	Ref.	Ref.	Ref.	Ref.
Dually eligible – full	37.95	18.73 (17.08-20.37)	20.79 (19.14-22.43)	16.58 (15.01-18.15)	15.97 (14.43-17.51)	16.07 (14.53-17.60)
Dually eligible – partial	33.68	14.46 (13.26-15.66)	14.03 (12.86-15.20)	8.30 (7.17-9.44)	7.62 (6.49-8.75)	7.51 (6.37-8.64)

Model 0: generalized linear model, zero-inflated negative binomial, or negative binomial model with no covariate adjustment;
Model 1: model 0, with further adjustment for age, gender, and race/ethnicity;
Model 2: model 1 with further adjustment for other patient characteristics;
Model 3: model 2, with further adjustment for SNF characteristics;
Model 4: model 3, with further adjustment for market competition, rural/urban locat

© 2020 Li Y et al. *JAMA Network Open.*

eReferences.

1. Temkin-Greener H, Campbell L, Cai X, Hasselberg MJ, Li Y. Are Post-Acute Patients with Behavioral Health Disorders Admitted to Lower-Quality Nursing Homes? *Am J Geriatr Psychiatry.* 2018;26(6):643-654.
2. CMS. *Centers for Medicare and Medicaid Services. Design for Nursing Home Compare Five-Star Quality Rating System, Technical users' guide. April 2018. Available at https://www.cms.gov/Medicare/Provider-Enrollment-and-Certification/CertificationandComplianc/downloads/usersguide.pdf.*
3. Abt. *Abt Associates Inc. Nursing Home Compare quality measure technical specifications. Available at https://www.cms.gov/Medicare/Provider-Enrollment-and-Certification/CertificationandComplianc/Downloads/New-Measures-Technical-Specifications-DRAFT-04-05-16-.pdf. April 4, 2016.*
4. Kimball CC, Nichols CI, Nunley RM, Vose JG, Stambough JB. Skilled Nursing Facility Star Rating, Patient Outcomes, and Readmission Risk After Total Joint Arthroplasty. *J Arthroplasty.* 2018;33(10):3130-3137.
5. Dejong G, Horn SD, Smout RJ, Tian W, Putman K, Gassaway J. Joint replacement rehabilitation outcomes on discharge from skilled nursing facilities and inpatient rehabilitation facilities. *Arch Phys Med Rehabil.* 2009;90(8):1284-1296.
6. Li Y, Glance LG, Yin J, Mukamel DB. Racial disparities in rehospitalization among medicare patients in skilled nursing facilities. *American Journal of Public Health.* 2011;101(5):875-882.
7. Li Y, Cai X, Glance LG. Disparities in 30-Day Rehospitalization Rates Among Medicare Skilled Nursing Facility Residents by Race and Site of Care. *Med Care.* 2015;53(12):1058-1065.
8. Finkelstein A, Ji Y, Mahoney N, Skinner J. Mandatory Medicare Bundled Payment Program for Lower Extremity Joint Replacement and Discharge to Institutional Postacute Care: Interim Analysis of the First Year of a 5-Year Randomized Trial. *JAMA.* 2018;320(9):892-900.
9. Zuckerman RB, Wu S, Chen LM, Joynt Maddox KE, Sheingold SH, Epstein AM. The Five-Star Skilled Nursing Facility Rating System and Care of Disadvantaged Populations. *J Am Geriatr Soc.* 2019;67(1):108-114.
10. Chodosh J, Edelen MO, Buchanan JL, et al. Nursing home assessment of cognitive impairment: development and testing of a brief instrument of mental status. *J Am Geriatr Soc.* 2008;56(11):2069-2075.
11. Morris JN, Fries BE, Mehr DR, et al. MDS Cognitive Performance Scale. *J Gerontol.* 1994;49(4):M174-182.
12. Li Y, Cai X, Harrington C, et al. Racial and Ethnic Differences in the Prevalence of Depressive Symptoms Among U.S. Nursing Home Residents. *J Aging Soc Policy.* 2018:1-19.
13. Saliba D, DiFilippo S, Edelen MO, Kroenke K, Buchanan J, Streim J. Testing the PHQ-9 interview and observational versions (PHQ-9 OV) for MDS 3.0. *Journal of the American Medical Directors Association.* 2012;13(7):618-625.
14. Belanger E, Thomas KS, Jones RN, Epstein-Lubow G, Mor V. Measurement validity of the Patient-Health Questionnaire-9 in US nursing home residents. *Int J Geriatr Psychiatry.* 2019;34(5):700-708.
15. Williams RL. A note on robust variance estimation for cluster-correlated data. *Biometrics.* 2000;56(2):645-646.
16. Greene WH. *Econometric analysis.* Upper Saddle River: Prentice Hall; 2001.

© 2020 Li Y et al. *JAMA Network Open.*
